# New image colocalization coefficient for fluorescence microscopy to quantify (bio-)molecular interactions

**DOI:** 10.1111/jmi.12008

**Published:** 2013-03

**Authors:** HD Herce, CS Casas-Delucchi, MC Cardoso

**Affiliations:** * Department of Biology, Technische Universität DarmstadtDarmstadt, Germany; ‡ Instituto de Física de Líquidos y Sistemas Biológicos (CONICET)La Plata

**Keywords:** Confocal Microscopy, Colocalization, Fluorescent Microscopy, Biomolecules, Molecular Interactions, High resolution microscopy

## Abstract

The spatial relationship, or degree of colocalization, between two or more types of molecules in live cells is commonly detected using fluorescence microscopy. This spatial distribution can be used to estimate the interaction between fluorescently labelled molecules. These interactions are usually quantified by analysing the correlation and/or the overlap between images, using the Pearson's and Manders’ coefficients, respectively. However, the correlation and overlap coefficients are parameters not designed to quantify molecular interactions. Here we propose a new colocalization coefficient specifically designed to quantify the interactions between molecules. In well-defined thermodynamic ensembles, this coefficient can in principle be used to calculate relevant statistical thermodynamic quantities such as binding free energies.

## Introduction

A fundamental task in common fluorescence microscopy is to infer from a single or a collection of images the underline interactions between fluorescently labelled molecules. In biology, for example, this could be the interaction between proteins. These interactions are usually enhanced or reduced by several factors, such as posttranslational modifications, changes in the expression levels of interaction partners, drugs designed to target these interactions, etc. To gain a deeper insight into these biological processes is of fundamental importance to quantify these interactions. This can be accomplished by analysing the relative spatial distribution between the molecules from the fluorescent images, a procedure termed colocalization analysis (Demandolx & Davoust, 1997, Costes *et al.*, 2004, Bolte & Cordelieres, 2006, Zinchuk *et al.*, 2007, Zinchuk *et al.*, 2011).

Colocalization analysis requires the simultaneous or serial detection of two or more fluorescently labelled molecules. The image obtained for each colour, or channel, is stored as three-dimensional arrays in which two of the three dimensions store the pixel position and the other dimension stores the fluorescence intensity. To characterize the interaction between fluorescently labelled molecules several parameters have been developed commonly called colocalization coefficients. Their usefulness depends on several factors such as the underlying biological process of interest, the noise level of the images and the information that needs to be extracted. To study interactions that require a resolution below the diffraction limit several methods based on Förster (or Fluorescence resonance energy transfer (FRET) have been developed (Pawley, 2006). Fluorescence resonance energy transfer-based methods can be used to study only interactions of pair of molecules below the diffraction limit, in a range of 1 to 10 nm. However, biological interactions can expand a much broader range and in many cases this high-resolution level is not necessary. For these cases the two most commonly used colocalization coefficients are the Pearson's coefficient (***R***) and the Manders’ coefficients (Manders *et al.*, 1992, Manders *et al.*, 1993). The first one is a measure of the local correlation between signals and the second is a measure of the overlapped fraction of each signal. Although we could intuitively expect that the correlation and overlap between the signals would increase if there is attraction, or decrease if there is repulsion between the molecules, this is not necessarily the case. The correlation and the overlap between images are useful tools to gain a qualitative idea of the underlying molecular interactions but they cannot be used to quantify these molecular interactions.

**Fig. 1:**
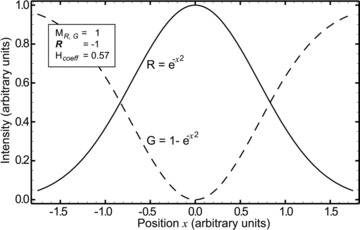
Intensity or spatial distribution of two kinds of particles, R and G, in a one-dimensional space. These distributions indicate a degree of repulsion between particles R and G. Both Manders’ coefficients are 1, indicating that the overlap between the two signals is complete. The coefficient ***R*** indicates that there is absolute anticorrelation between the two signals. The *H_coeff_* indicates that the particles repel each other and this repulsion can still be bigger even when there is anticorrelation between the signals.

We can use a simple one-dimensional example to show that correlation and overlap are not reliable, or not well-defined parameters to quantify molecular interactions. Imagine two densely packed kinds of molecules, where the instantaneous distribution of each molecule type is given by the spatial distribution functions shown in Figure 1. This could be visualized as two kinds of interacting liquids in a thin tube. These distributions show clearly a degree of repulsion between particles R and G and that the strength of the repulsion between these two types of molecules can still be higher, in a more extreme case of repulsion the molecular distributions would not overlap. However, the Manders’ coefficients reach their maximum value, indicating that the overlap between the two signals is complete. The Pearson's coefficient indicates that there is absolute anticorrelation between the two signals. Both results correctly describe the overlap and the correlation between these two types of molecules but these parameters clearly cannot be used to quantify the underlying interaction between the molecules. For example, the repulsion between the two types of molecules can still be stronger leading to a stronger separation between the two molecules. However, ***R*** has already reached a maximum value and would not recognize this difference. In other words, ***R*** is indicating that there is repulsion but cannot quantify the strength of the repulsion.

**Fig. 2:**
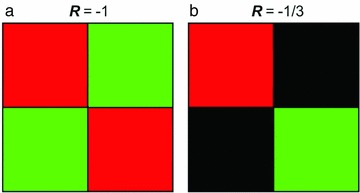
Two images were the red and green signals mutually exclude each other. In (a) the two signals cover in equal amounts the whole image while in (b) there are empty pixels. In (b) although the images do not colocalize ***R*** is more than -1.

Here we propose a colocalization coefficient that characterizes the interaction between molecules inspired in the following molecular-based picture: if the intensity *I* in the pixel of an image is proportional to the number of labelled molecules in that region, then the probability to find a molecule *r* in the same pixel as a molecule *g* can be obtained from an image by,


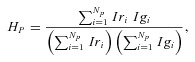
(1)

where *Ir_i_* and *Ig_i_* is the intensity of the channels *r* and *g* in the pixel *i* and *N_p_* is the total number of pixels. If the molecules do not interact, and are therefore randomly distributed over the available number of pixels *N_p_*, then *H_p_= 1/ N_p_*. This last value can help to stress the interpretation of *H_p,_* i.e. if the particles are randomly distributed then the probability to find a given particle *r* colocalizing with, or at the same pixel *i* of, a given particle *g* is inversely proportional to the total number of pixels and this particular result is independent of the number of particles r and g. Usually, in biology, it is of interest to compare colocalization results of molecules distributed in different cells that exhibit high morphological variability. Therefore, it is reasonable to normalize this probability relative to its random distribution,


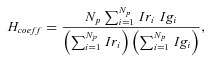
(2)

in this way when the particles do not interact, or are randomly distributed, then *H_coeff_= 1*. More strictly, this normalization should be done by multiplying *H_p_* by the area of integration. In the case of images this is proportional to *N_p_*, but other normalization conditions can be implemented accordingly depending on the case. We can see in the example shown in Figure 1 that this coefficient has a value somewhere between 0 and 1, less than the random value of 1, indicating that there is repulsion between the particles. However, this repulsion can still be higher such that there could be complete mutual exclusion, in this case it would be *H_coeff_= 0*. The coefficient ***R*** reaches a minimum because, although the repulsion between the particles could have a higher strength, the signals are already anticorrelated. It should be emphasized that even when the particles could have absolute mutual exclusion, this does not necessarily mean that ***R*** will be equal to *–1*. This can be visualized with a simple example shown in Figure 2. In both cases, Figures 2 (a) and (b), the red and green signals do not colocalize. However, only for case (a) ***R*** is –1 while in the case (b) ***R*** is –1/3. The reason can be understood if we keep in mind that ***R*** is a measure of correlation and in (b) there are pixels that are not being occupied by any of the two signals. This fact enhances the correlation between the signals (i.e. both signals are excluded simultaneously from the empty pixels) although the signals, or molecules, themselves do not colocalize.

A fundamental reason why *H_coeff_* works better to quantify interactions can be understood by looking at it from a statistical thermodynamic point of view. In statistical thermodynamics the fundamental parameter used to calculate interactions between molecules is the probability distribution of the molecules. The actual strength of the interactions can be calculated from this parameter using different equations where the specific form will depend on the particular thermodynamic ensemble. In this way, the *H_coeff_* can be used to numerically calculate the interactions in well-defined thermodynamic ensembles. Although, live biological systems are clearly out of thermodynamic equilibrium, several processes can still be well approximated within certain conditions with a given thermodynamic ensemble. For example, to study the spontaneous binding of two proteins in a cell in several cases it can be assumed that within the average binding time, the volume, temperature and number of molecules remains constant and therefore the system can be assumed to be approximately in a canonical ensemble. In this case the free energy of the system is given by the Helmholtz free energy. In this regime, the *H_coeff_* could be used to estimate the relative Helmholtz-free energy, *F*, or the average work required to get the two molecules in the same pixel using,


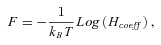
(3)

where *k_B_* is the Boltzmann constant and T the temperature of the system. This allows connecting this particular colocalization coefficient with well-defined thermodynamic quantities.

In the example shown in Figure 1, if the system would also be in a canonical ensemble this would mean that the Helmholtz-free energy can be used to calculate the interactions between these two types of molecules. Furthermore, we will show that this coefficient can in principle also be used to calculate several thermodynamics parameters in other thermodynamic ensembles such as the grand canonical ensemble. This can be achieved through a simple generalization of this coefficient and using the Kirkwood-Buff theory (Kirkwood & Buff, 1951).

Next we present and characterize the *H_coeff_* as a colocalization coefficient. We compare it with other well-established colocalization coefficients using simple examples to characterize the cases in which each coefficient could be useful. We discuss the behaviour of each coefficient when different sources of noise are present. Finally, we apply the *H_coeff_* to analyse the colocalization between sites of replication and particular chromatin regions along the cell cycle, using the *H_coeff_* and ***R*** coefficients to analyse biological images with different signal-to-noise ratios.

### Description of commonly used colocalization coefficients

The coefficient ***R***, the overlap coefficient (*Over*) and the Manders’ coefficients (*M_R_* and *M_G_*) are widely used to measure the degree of correlation or overlap between two signals (Manders *et al.*, 1993, Zinchuk *et al*., 2007, Zinchuk & Grossenbacher-Zinchuk, 2009). Among them, ***R*** is the oldest, best characterized and most commonly used,


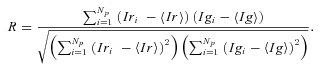
(4)

This coefficient measures the correlation between two signals, in this case *Ir_i_* and *Ig_i_*. As we showed, if particles r and g interact, they will display a correlated signal. However, as mentioned before, signal correlation is not sufficient to describe the strength of the interaction between particles.

The Overlap coefficient can be obtained from ***R*** by removing the average values,


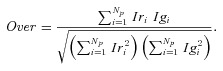
(5)

A disadvantage of this coefficient is that it does not have a clear interpretation such as ***R*** and the Manders’ coefficients.

The Manders’ coefficients have a clear interpretation and characterize very well the fraction of overlapped signal of each channel. The Manders’ coefficients have been introduced to solve the strong influence that the ratio of the number of objects in each signal channel has over ***R*** and the Overlap coefficients. To solve this, the degree of colocalization is expressed separately for each channel as,


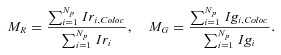
(6)

These coefficients are clearly much better for characterizing the overlap between the signals than the Overlap coefficient. If particles r and g attract each other, it is expected that the signals will display a higher overlap. However, if the overlap is high, this does not necessary imply any underling attraction between the particles, as showed in the example of Figure 1.

Each coefficient has a different range of values that characterize different regimes. In the case of ***R***, if the signals are strictly correlated then ***R***=*1*, if the images are strictly uncorrelated then ***R***=*-1*, and if there is no correlation, or random correlation, between the signals ***R***=*0*. The Overlap and Manders’ coefficient have only two characteristic values, 1 if the images fully overlap, and 0 if there is no overlap. Similarly to ***R***, the *H_coeff_* also has three characteristic regions of values. The difference is that the *H_coeff_* characterizes interactions. If the particles repel each other it is expected that *H_coeff_ <1*, if the particles do not interact and therefore the signals are randomly distributed *H_coeff_= 1*, and if the particles attract each other *H_coeff_ >1*. In particular, if there is no overlap between the signals *H_coeff_= 0* and if both signals are concentrated in a single pixel *H_coeff_*=*N_p_*.

The *H_coeff_*, ***R***, and *Over* coefficients implicitly do not exclude any part of the image under consideration. However, the Manders’ coefficient *M_R_* (*M_G_*) implicitly excludes from the computation any region of the image where the R (G) signal is zero. This implicit exclusion can be particularly useful to narrow the region of interest. The other aspect of interest is that the equations listed only consider the case of the interaction of two signals or molecules but in some cases it is of interest to study simultaneously the binding or colocalization of more than two molecules labelled with different dyes. Therefore, we discuss in the Supplementary material, part (i) and (ii), possible generalizations of the *H_coeff_* and ***R*** to consider these cases. The results presented in Tables 1–3 are expanded in Tables 1–3 of the supplementary material to include these parameters.

**Table 1:** Average colocalization coefficients for R (red) particles and G (green) particles located at 4 and 9 discrete positions. In (a)–(d), (i), (j) we consider 4 R and 3 G particles, and in (e)–(h) 4 R and 4 G particles. In (a) and (e) all the particles are randomly distributed. (b) One G particle is bounded to one R particle. (c) It is considered two R particles individually bound to two G particles and two R particles bound to a single G particle. In (d) and (h) all particles are bound. (f) Each R particle is individually bound to a G particle. (g) It is considered 3 R and 3 G particles all bound together and 1 R bounded to 1 G. In (i) and (j) it is considered the case of repulsion, as indicated by the arrows, between R and G particles. Herce et al. 2012

	nr and ng	*N_p_*	*H_coeff_*	*R*	*Over*	*M_R_*	*M_G_*
(a)	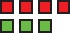	4	1	0	0.571	0.578	0.685
		9	1	0	0.317	0.297	0.375
(b)	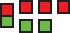	4	1.25	0.250	0.688	0.686	0.789
		9	1.667	0.278	0.508	0.473	0.583
(c)	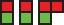	4	2	0.906	0.965	1	1
		9	3.667	0.934	0.953	1	1
(d)	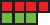	4	4	1	1	1	1
		9	9	1	1	1	1
(e)	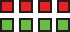	4	1	0	0.606	0.685	0.685
		9	1	0	0.351	0.375	0.375
(f)	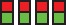	4	1.75	1	1	1	1
		9	3	1	1	1	1
(g)	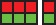	4	2.875	1	1	1	1
		9	6	1	1	1	1
(h)	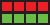	4	4	1	1	1	1
		9	9	1	1	1	1
(i)	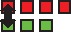	4	0.916	−0.084	0.532	0.542	0.648
		9	0.916	−0.035	0.294	0.275	0.349
(j)	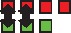	4	0.833	−0.166	0.493	0.507	0.613
		9	0.833	−0.069	0.270	0.253	0.323

**Table 2:** Colocalization coefficient results obtained after different combinations of abstract images composed of equal objects. In the first two rows are shown the pair of images taken as the red (R) and green (G) channel and in the rest of the rows the colocalization coefficients for each pair of images. Herce et al. 2012

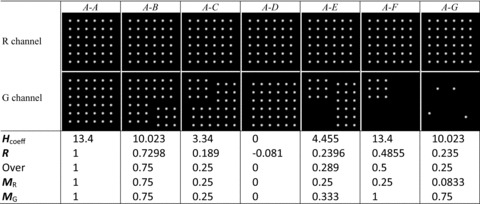

**Table 3:** Colocalization coefficient results obtained after different combinations of abstract images composed of different objects. In the first two rows are shown the pair of images taken as the red (R) and green (G) channel and in the rest of the rows the colocalization coefficients for each pair of images. Herce et al. 2012

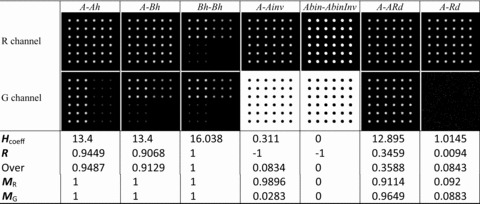

The colocalization coefficients considered so far compare signals at each individual pixel. In the case of the *H_coeff_*, the result can be interpreted as a magnitude that is proportional to the probability of finding a given particle R in the same pixel as a given particle G. Analogously, in the case of ***R***, the results can be interpreted as the average correlation of the signal R and the signal G at each pixel. This analysis does not provide information about interactions or correlations between signals that go beyond one pixel. This can be critical if we consider the interaction or correlation between molecules mediated by other molecules. In this case if molecules within a region do not directly interact, the colocalization analysis could wrongly indicate that the signals are not correlated. To take into account this possibility we consider next a spatial generalization of the colocalization coefficients.

### Spatial correlation

Studding the spatial correlation between images has a wide spectrum of applications. This can be used to obtain relevant structural information to further characterize the interaction between molecules. This structural information can also be used to align images and reduce noise (Wu *et al.*, 2010, Zinchuk *et al*., 2011). In biology, there are several cases in which different molecules could interact with each other but still be separated by a distance greater than the pixel size. There are several important biological examples in which the interaction between molecules is mediated by other intermediate molecules along a given pathway. For example an extracellular signalling protein can bind to a receptor located at the cell plasma membrane and activate a pathway leading to the translocation of a cytosolic protein to the nucleus. In this example, the interaction between the signalling protein and the cytosolic one is mediated by membrane receptors and other downstream proteins that lead to the translocation of the cytosolic protein into the nucleus. This is the case for example of the nerve growth factor that binds to TrkA receptors leading to the nuclear translocation of protein kinases such as mitogen-activated protein kinase (Sorkin & Von Zastrow, 2002).

In these cases it is necessary to evaluate these coefficients between particles located in different pixels and in this sense colocalization can be thought as a special case of spatial correlation. The spatial generalization of the coefficients can be done as follows, for the *H_coeff_*


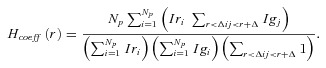
(7)

and for ***R***


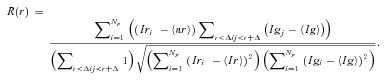
(8)

Where Δ is the thickness of the interval evaluated at a distance *r* and *Δ_ij_* is the distance between the pixel *i* and pixel *j*. The extra lower summation is used to normalize the equation by the number of pixels counted in the g channel for each distance *r*. The spatial generalization of the other coefficients can be done in a similar way.

The spatial generalization of the *H_coeff_*, Eq. 7, provides a natural connection with the Kirkwood–Buff theory (Kirkwood & Buff, 1951), which is the most powerful theory of solution. This theory only requires the knowledge of integrals over the distance of radial distribution functions, to calculate thermodynamic properties such as compressibility, partial molar volumes and chemical potentials.

Next we present three applications of the colocalization coefficients. First, we will consider a couple of simple abstract examples, one that resembles interacting particles and another one for abstract images. Finally, we will show and discuss the results of these coefficients on biological images. To these biological images we will also apply the two spatial correlation coefficients.

### Application to simple abstract models of interacting particles

To gain more insight into the colocalization coefficients, we present now a simple example that mimics interactions between molecules and where the average quantities for all the parameters can be exactly evaluated. In this example, we consider two kinds of particles labelled in red and in green, which are free to move between the pixels, the area of each pixel is assumed to be big enough to hold all the particles.

The interaction between particles can be either: (a) Two or more particles can be absolutely bound and always colocalize. (b) Repel each other such that there is absolute exclusion between two particles, being always in different pixels. (c) No interaction, and the particles move randomly relative to each other. Based on these three kinds of interactions, we constructed different cases and computed the average value for each coefficient over all the possible configurations for each case. We computed these average values over all possible conformations for images composed of four and nine pixels. Similarly, in biological studies, one or several images are also used to obtain statistical relevant information.

In (a) the particles do not interact and therefore there is a random distribution over the pixels. The *H_coeff_* and ***R*** all have well-defined values; this is not the case with the Overlap and Manders’ coefficients. In (b) a red and a green particle are bound. In this case, the probability to find a green particle and red particle in the same pixel increases and, as expected, there is an increase in all coefficients relative to their value for the random distribution (a). In (c) every green particle is bound to at least one red particle and we can see that the Manders’ coefficients reach their maximum value while the rest coefficients only reach this maximum value when all particles are bound together as in case (d). In (e) there is an extra G particle relative to (a), and there are equal number of particles of each type. In this case the value of *H_coeff_* and ***R*** do not differ, each coefficient indicates that the particles do not interact and are not correlated respectively. This information is more difficult to obtain using both Manders’ and the Overlap coefficients.

If the numbers of particles are the same ***R***, Overlap and Manders’ do not distinguish between cases (f), (g) and (h). The reason is that in these cases the signals will be absolutely correlated and overlapped. Since the *H_coeff_* measures the probability to find an R and a G particle at the same position relative to the same probability when the particles do not interact and are randomly distributed, its maximum can only be reached when all the particles are bound together as in case (h).

We can see that when the particles repel each other all coefficients decrease relative to their random values. In the case of ***R*** and the *H_coeff_* this random value is 0 and 1, respectively, independently of the problem.

Colocalization coefficients, besides being commonly used to study biological molecules through the analysis of images, are also commonly used to compare the degree of similarity between images. Therefore, next we will consider the evaluation of the coefficients on abstract images as was done originally by Manders (Manders *et al.*, 1993).

### Application to simple abstract images

We can obtain a further insight into the features of each parameter by looking at abstract images. In Table 2, we use similar images as the ones introduced by Manders (Manders *et al.*, 1993), which contain patterns of objects defined by Gaussian intensity distributions. Each Gaussian object in the image has a diameter of 12 pixels full with at half maximum. Each image contains 256 × 256 pixels. In Table 3 we also considered images with other objects, such as Ah and Bh where the objects have the same shape but with a different intensity, Ainv is image A inverted, Abin and Abin inverted are binary images, ARd is image A with added Gaussian noise, and Rd a randomly generated image.

We can see in Table 2 A-A, that when the two images are the same, and therefore, the signals are perfectly correlated and overlapped the ***R***, *M_R_*,_G_, and Over reach their maximum values. The *H_coeff_* is bigger than 1, indicating that if the images are showing two kinds of particles then these particles attract each other. This attraction could still be higher if all the particles would be concentrated in the same pixel and this is why the *H_coeff_* still does not reach its maximum value.

In A–B, the numbers of Gaussian objects in B are the same as in A, but only 75% of them overlap with the Gaussian objects in A. This reduces the correlation and therefore ***R*** and reduces all overlap coefficients to 0.75. This also reduces the *H_coeff_* since this signal would indicate that there is less probability that a given particle R would be in the same pixel as a given particle G. In A–C, the amount of Gaussian objects in C are the same as in A but only 25% of them overlap with the Gaussian objects in A. This further reduces the correlation and therefore ***R*** and all overlap coefficients to 0.25. This accordingly reduces the *H_coeff_*.

In A–D, there is no overlap between the Gaussian objects in the images. This results in ***R*** being close to 0 that could be wrongly interpreted as a random correlation between the objects. If these objects would represent particles and the image would contain a statistical significant number of these particles, then ***R*** might seem to indicate that there is no interaction between the particles. However, if the particles were not able to overlap this would indicate that there is repulsion between them. This apparently contradictory effect is simple to understand if we keep in mind that ***R*** measures correlation and that correlation cannot be strictly associated with interactions. In this case, although the Gaussian objects in the two channels are not correlated giving negative contributions to the ***R***, in the image there are also significant regions where there are simultaneously no objects in both channels, adding a significant positive correlation. These two contributions compensate in this case, resulting in an overall almost 0 value for ***R***. A simpler example was presented in Figure 1. This clearly does not mean that the objects are randomly distributed. If the objects would represent labelled molecules, the image would indicate that they repel each other. Otherwise, at least some of the objects would overlap. Therefore, this example demonstrates that correlation should be used very carefully when it is used to parameterize the interaction between labelled molecules from images. The *H_coeff_* would indicate that there is absolute repulsion between the objects since there is no overlap between them.

In A–F, the two images have different number of objects but all the objects in the G channel colocalize with the objects in the R channel. In this case the *H_coeff_* indicates that the relative interaction between the particles captured in each channel is the same as in the case A-A. However, the correlation and overlap between the signals is reduced and therefore all the other coefficients are reduced relative to the case A-A.

In Table 3 A-Bh, the number of objects are the same but in the G channel the objects have the same Gaussian distribution but their intensity is four times more intense in the first quadrant composed of nine objects in the upper left relative to the quadrant in the lower left and it is gradually reduced from left to right. If these signals can be interpreted as interacting molecules, this conformation would indicate that it is equally probable to find an R particle with a G particle than in the case A-A. This would indicate that the attraction between particles in both channels is the same as in the case AA. However, the correlation between the channels is reduced and ***R*** has a lower value.

In Bh-Bh, both signals are more concentrated indicating an increased probability to find an R particle with a G particle and this is reflected in an increase in the *H_coeff_*.

In A-Ainv, the G channel is the inverted image of the R channel. This case is analogous to the one-dimensional example shown in Figure 1. If each channel can be interpreted as interacting molecules, then this will indicate that there is repulsion between the molecules. However, since there is a partial overlap between both channels this repulsion is not absolute, particles repel but thermal fluctuations for example could partially break the energetic barrier imposed by this repulsion producing partial overlap. Although the repulsion is not absolute, the signals are absolutely anticorrelated bringing ***R*** to its minimum. Furthermore, in this case the R channel is almost completely overlapped with the G channel. Therefore, Manders’ coefficient shows that the R channel fully overlaps with the G channel. However, this result also cannot be linked to the underlying molecular interactions.

In Abi-Abii, the signals of both channels do not overlap. This case would indicate an absolute repulsion between the molecules of each channel. Therefore, the probability of finding a particle in the R channel colocalizing with a particle in the G channel is zero and the *H_coeff_* is zero. In terms of the free energy, this would indicate that the energetic cost of bringing the particles of each channel to the same pixel is infinite. For example, if the system is in a canonical ensemble then the free energy is proportional to the logarithm of the *H_coeff_* and in this case this value would be infinite.

In A-Rd, the G channel is randomly distributed relative to the R channel indicating that there is no interaction between the particles of each channel. This brings the *H_coeff_* close to one and, since there is also no correlation, ***R*** is close to zero.

In this last case a Gaussian noise was added to the image. Further, in microscopy images there are several other sources of noise that could make more difficult to interpret the results. Therefore, next we will use a pair of these abstract images to characterize the behaviour of each parameter as some of these sources of noise are progressively increased.

### Noise analysis

Several sources of noise are present in standard fluorescence microscopy images, which affect the colocalization coefficients in different ways. To simulate the sources of image deterioration that commonly appear in confocal imaging and look at the effect on the colocalization parameters, images were gradually degraded with background, crosstalk and Poisson noise.

### Homogeneous background

**Fig. 3:**
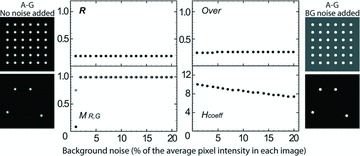
Colocalization coefficients dependence on added homogeneous background noise (BG) on the pair of images in Table 2 column A-G. In the first and last columns, the images without any noise addition and with the addition of 20% background noise are presented, respectively. In the plots of each coefficient, the background noise is linearly increased between 0 and 20% of the average pixel value of each image.

This kind of noise is usually generated by ‘dark current’ (DC), which is a relatively small electric current that flows through the photo multipliers even when no photons are entering the device. This can be simulated by,


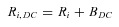
(9)

and


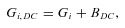
(10)

where *B_dc_* is a constant value that represents the uniform background. For the R and G channels we choose the pair of images in Table 2 column A-G. In Figure 3 we show the result for a variable background noise, which is linearly increased between 0 and 20% of the average pixel value of each image.

We can see that this kind of noise has the strongest effect on Manders’ since it increases drastically the overlap between the images. The coefficient ***R*** and Overlap values remain almost constant and the *H_coeff_* has an effect that is proportional to the added noise.

### Crosstalk

**Fig. 4:**
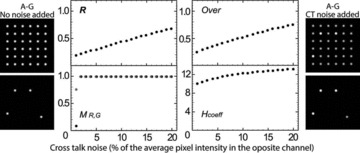
Colocalization coefficients dependence on added crosstalk noise (CT) on the pair of images in Table 2 column A-G. In the first and last columns the images without any noise addition and with the addition of 20% noise of the average pixel signal value in the opposite channel are shown. In the plots of each coefficient, the crosstalk noise is linearly increased between 0 and 20% of the average pixel signal value in the opposite channel.

This kind of noise could be caused by cross-reactivity of the fluorescent probes and signal leak between channels produced by an inappropriate filtering of the signal by the optical components (Manders *et al.*, 1993, Gavrilovic & WÄHlby, 2009). This kind of noise can be simulated using


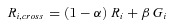
(11)

and


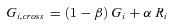
(12)

where α and β represent the crosstalk factors. In this case, we varied α and β such that the crosstalk in each pixel was between 0 and 20% of the pixel intensity value in the opposite channel (Fig. 4).

There is a strong effect on all the coefficients except on the *H_coeff_* where the change is proportional to the crosstalk noise added to the images.

### Poisson noise

The major source of noise in a confocal image is usually quantum noise, originated mainly by statistical quantum fluctuations of the number of photons detected. This kind of noise can be simulated using a Poisson distribution,


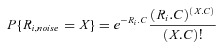
(13)

and


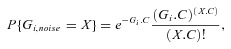
(14)

where *P* is the probability that a grey value of the image with the added noise is equal to *X*. The number of simulated photons per grey-value is represented by *C*.

**Fig. 5:**
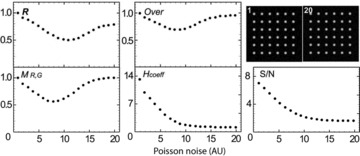
Colocalization coefficients values after independently adding Poisson noise on the pair of images in Table 2 column A-A, and then progressively decreasing the threshold in each of them. The images with the highest threshold (1) and the lowest threshold (20) considered are presented, as well as the signal to noise (S/N) ratio for each threshold considered.

Instead of variably adding noise, we degraded independently the images adding Poisson noise in a signal to noise ratio of 1 db. Then we applied a threshold to the signal and we gradually decreased it. In this case we compared two independently degraded images generated from Table 2 column A-A. In Figure 5 we can see these images at the initial and maximum cutoff considered in position 1, and the minimum cutoff value considered at position 20.

An interesting aspect of this kind of noise is that ***R***, the overlap and the Manders’ coefficients all increase, after a certain value of increasing noise, indicating higher correlation and overlap. The *H_coeff_* still decreases progressively to a random value. We will show next that in real images obtained from biological samples a similar effect is takes place.

Next, we consider the behaviour of ***R*** and the *H_coeff_* on confocal images obtained from actual biological samples considering two levels of thresholding to increase the signal to noise ratio.

### Biological application to cell-cycle analysis

Before a cell can divide, its DNA must be completely duplicated, a process called DNA replication, which happens during the synthesis (S-) phase of each cell cycle. DNA replication takes place in a defined spatiotemporal order. In general, euchromatic, gene-rich, domains reside in the interior of the nucleus and replicate in early S-phase, whereas the gene-poor heterochromatin occupies clustered domains and replicate mostly in late S-phase (Leonhardt *et al.*, 2000, Casas-Delucchi *et al.*, 2011a, Casas-Delucchi *et al.*, 2011b).

**Fig. 6:**
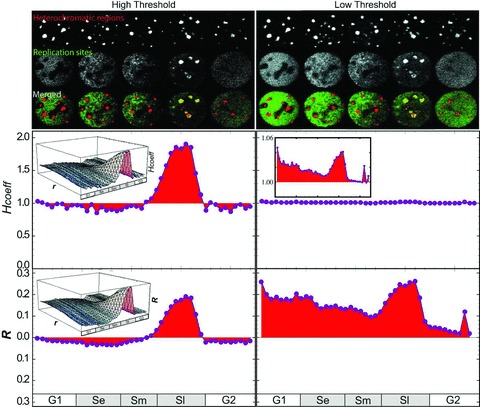
Application of the *H_coeff_* and ***R*** to biological samples and impact of thresholding. In the first three rows confocal time lapse microscopy images of the same cell at different stages of the cell cycle are shown. The heterochromatic regions are labelled in red and the replication sites are labelled in green. For details on the labelling and acquisition procedures see (Casas-Delucchi *et al*., 2011b). The first five columns depict the images after applying a high threshold. The rest of the columns show the same images but with a low threshold. The 4^th^ and 5^th^ rows display the *H_coeff_* and ***R*** results for 40 images acquired throughout the cell cycle at 30-minute intervals. The left insets present the spatial correlation functions obtained using Eqs. (7) and (8), where the axes ***r*** is the distance between pixels and in the figure is shown the correlation up to a distance radius of 20 pixels. The right inset shows an amplified view of the plot for the *H_coeff_*. The software used to perform these analyses has been made open source and can be downloaded from http://cardoso-lab.org/pages/software.htm).

In Figure 6 sites of active replication are labelled in green and heterochromatic regions, also called chromocentres, are labelled in red. The images were acquired following a single cell along the cell cycle as described in Casas-Delucchi, van Bemmel *et al.* 2011. To reduce the background noise a local threshold was applied. This local threshold consisted on calculating the average intensity on a square patch of 40 pixels around each pixel was measured and if the pixel intensity was less than 1.2 (high threshold), or 1.0 (low threshold), times higher than this average the pixel was made equal to zero. The software we developed to perform these calculations can be downloaded from http://cardoso-lab.org/pages/software.htm).

We can see that in early S-phase there is anticorrelation between the two signals indicating that the replication is occurring mostly outside of the heterochromatic regions. However, in the late S-phase, as the replication foci accumulate in the heterochromatic regions, the colocalization sharply increases. The coefficients ***R*** and *H_coeff_* qualitatively agree. However, if we lower the threshold of the images, the colocalization according to ***R*** increases significantly while the *H_coeff_* gets closer to 1. This indicates that, although the signals can be more correlated according the ***R***, the *H_coeff_* correctly indicates that the signals are more randomly distributed when the threshold is reduced. This behaviour is similar to the one shown in Figure 5 for the Poisson noise that enhances the correlation between the signals when the noise increases. In the inset we show the spatial correlation, Eqs. (7) and (8), between the signals. In this case, the spatial correlation of the replication sites relative to the heterochromatic regions is presented. As the distance to the heterochromatic regions increases the correlation between replication sites relative and heterochromatic regions approximates to 1 in the case of the *H_coeff_* and to 0 in the case of ***R*** indicating that the signals become randomly as the distance between the two signals increases.

## Conclusions

The interaction between molecules is a fundamental parameter of interest in science. Here, we showed that in microscopy image analysis this parameter can be properly quantified using the *H_coeff_*. Furthermore, in a well-defined thermodynamic ensemble this coefficient can in principle be used to calculate thermodynamic quantities such as binding free energies. We generalized this analysis to not only the time dimension but in particular to the spatial dimension.

To illustrate the behaviour of the *H_coeff_* and other commonly used colocalization coefficients under different conditions, we presented two simple examples in detail. Analysing all the possible configurations of a simple example of interacting molecules, we were able to show that indeed the *H_coeff_* is the only coefficient that has a proper range to quantify interactions between molecules. Analysing abstract images we could see that even if the *H_coeff_* is specially suited to quantify interactions, it can also complement the information provided by ***R*** and the Manders’ coefficients to compare images. Each coefficient quantifies colocalization by measuring complementary quantities, a measure of the strength of the interaction between the molecules can be obtained by the *H_coeff_*, a measure of the correlation between the signals can be obtained using the ***R***, and a the degree of overlap between the signals can be obtained using the Manders’ coefficients.

Several sources of noise are always present in the acquisition of microscopy images. Therefore, we analysed the individual effect of the most common sources of noise on the colocalization coefficients. We found that, as expected, background noise and crosstalk have the strongest effect on Manders’ coefficient. Background noise has no effect on the ***R***. This can be understood if we keep in mind that a constant addition in the signal does no change the relative increase or decrease of a signal and therefore this cannot affect the correlation between signals. The most surprising result is the effect of the Poisson noise in the images. The correlation and the Manders’ coefficient initially decrease with increasing levels of noise and after a certain level the noise increases the correlation and the overlap between the signals. The *H_coeff_* instead shows a steadily decrease toward 1 as the signal to noise ratio decreases. We show that a similar effect is present as the threshold of microscopy images is decreased.

The *H_coeff_* and ***R*** can be naturally affected by the relative number of molecules captured in each channel. When there is a big difference between the numbers of molecules captured in each channel the coefficients could indicate no interaction or correlation even when the molecules might attract each other. This is not a problem for the Manders’ coefficients, which were specifically designed to correct this behaviour. Here, we also propose simple generalizations of the *H_coeff_* and ***R*** (such as *H_R,G_*, *H_RG_*, *P_R,G_* and *P_RG_*, see Supplementary material) to improve the behaviour of these coefficients when there is a big difference in the relative number of molecules in each channel.

The resolution of biological images has recently increased beyond the diffraction limit making possible to resolve individual molecules (Baddeley *et al.*, Grunwald *et al.*, 2008, Schermelleh *et al.*, 2008). In this case, even when molecules might not colocalize they might have a range of attraction or repulsion that goes beyond a single pixel. Here, we generalize the concept of colocalization to spatial correlation functions where colocalization is a particular case.

Finally, we applied the generalized *H_coeff_* and ***R*** to quantify the interaction between replication sites and heterochromatic regions along the cell division cycle. This shows a stronger attraction of the replication foci toward the chromocentres as the cell enters late S-phase. With low noise the *H_coeff_* and ***R*** qualitatively agree. However, when the noise is increased, ***R*** displays an artifactual higher colocalization of replication sites with the heterochromatic regions even in the G1 phase. This effect is consistent with the enhancement of the correlation between the images induced by Poisson noise as we simulated with model images. On the other hand, when the noise is increased the *H_coeff_* correctly describes a more random behaviour between the molecular signals.

## Supporting Information

**Fig. 1.** Qualitative representation of an image with two channels, red and green, and different possible regions of interest. (a) The region of interest is the whole image. (b) The region of interest is defined by the pixels where the signal of the red or green channel is higher than zero. (c) The region of interest is defined by the pixels where one of the signals is greater than zero, in this case the red signal. (d) The region of interest is defined as the pixels where both channels have a signal greater than zero.

## References

[b1] Baddeley D, Chagin VO, Schermelleh L (2010). Measurement of replication structures at the nanometer scale using super-resolution light microscopy. Nuc. Acids Res.

[b2] Bolte S, Cordelieres FP (2006). A guided tour into subcellular colocalization analysis in light microscopy. J. Microsc.

[b3] Casas-Delucchi CS, Brero A, Rahn HP, Solovei I, Wutz A, Cremer T, Leonhardt H, Cardoso MC (2011a). Histone acetylation controls the inactive X chromosome replication dynamics. Nat. Comm.

[b4] Casas-Delucchi CS, van Bemmel JG, Haase S (2011b). Histone hypoacetylation is required to maintain late replication timing of constitutive heterochromatin. Nuc. Acids Res.

[b5] Costes SV, Daelemans D, Cho EH, Dobbin Z, Pavlakis G, Lockett S (2004). Automatic and quantitative measurement of protein-protein colocalization in live cells. Biophys. J.

[b6] Demandolx D, Davoust J (1997). Multicolour analysis and local image correlation in confocal microscopy. J. Microsc.

[b7] Gavrilovic M, Wählby C (2009). Quantification of colocalization and cross-talk based on spectral angles. J. Microsc.

[b8] Grunwald D, Martin RM, Buschmann V, Bazett-Jones DP, Leonhardt H, Kubitscheck U, Cardoso MC (2008). Probing intranuclear environments at the single-molecule level. Biophys. J.

[b9] Kirkwood JG, Buff FP (1951). The statistical mechnical theory of solutions. I. J. Chem. Phys.

[b10] Leonhardt H, Rahn HP, Weinzierl P, Sporbert A, Cremer T, Zink D, Cardoso MC (2000). Dynamics of DNA replication factories in living cells. J. Cell Biol.

[b11] Manders EM, Stap J, Brakenhoff GJ, van Driel R, Aten JA (1992). Dynamics of three-dimensional replication patterns during the S-phase, analysed by double labelling of DNA and confocal microscopy. J. Cell Sci.

[b12] Manders EM, Verbeek FJ, Aten JA (1993). Measurement of co-localization of objects in dual-color confocal images. J. Microsc.

[b13] Pawley JB (2006). *Handbook of Biological Confocal Microscopy*.

[b14] Schermelleh L, Carlton PM, Haase S (2008). Subdiffraction multicolor imaging of the nuclear periphery with 3D structured illumination microscopy. Science.

[b15] Sorkin A, Von Zastrow M (2002). Signal transduction and endocytosis: close encounters of many kinds. Nat. Rev. Mol. Cell Biol.

[b16] Wu Y, Eghbali M, Ou J, Lu R, Toro L, Stefani E (2010). Quantitative determination of spatial protein-protein correlations in fluorescence confocal microscopy. Biophys. J.

[b17] Zinchuk V, Grossenbacher-Zinchuk O (2009). Recent advances in quantitative colocalization analysis: Focus on neuroscience. Prog. Histochem. Cytochem.

[b18] Zinchuk V, Wu Y, Grossenbacher-Zinchuk O, Stefani E (2011). Quantifying spatial correlations of fluorescent markers using enhanced background reduction with protein proximity index and correlation coefficient estimations. Nat. Protoc.

[b19] Zinchuk V, Zinchuk O, Okada T (2007). Quantitative colocalization analysis of multicolor confocal immunofluorescence microscopy images: Pushing pixels to explore biological phenomena. Acta Histochem. Cytochem.

