# Supplementary material for: New image colocalization coefficient for fluorescence microscopy to quantify (bio-)molecular interactions
Source: J Microsc. 2013 Mar;249(3):184–94. doi: 10.1111/jmi.12008 (PMC3599484; doi:10.1111/jmi.12008)
Supplement: Supplementary file 1 [file jmi0249-0184-SD1.pdf]

Supplementary material

New image colocalization coefficient for fluorescence microscopy to quantify interactions between (bio-)molecules

Henry D. Herce [1, 2, †], Corella S. Casas-Delucchi [1], and M Cristina Cardoso [1, †].

[1] Department of Biology, Technische Universität Darmstadt, 64287 Darmstadt, Germany.
[2] Instituto de Física de Líquidos y Sistemas Biológicos (CONICET), 59 N° 789, c.c. 565, 1900, La Plata.

[†] Corresponding author

(i) Regions of interest in image analysis and generalization of the H_{coeff} and *R*

In the analysis of images with two channels we can distinguish at least four general regions of interest that could be useful for different applications (Fig. 3): (a) the full image; (b) a region where both signals are bigger than zero, where zero intensity pixels are not counted in the analysis; (c) a region where one of the signals is higher than zero (either the red as marked in Fig (c) or the green; (d) the region where the signals overlap.

The coefficient ***R*** and the Overlap coefficients are usually used for the colocalization analysis in case (a). These two coefficients depend strongly on the ratio of molecules of each type. In cases where the relative ratio of molecules of each type differs strongly it is qualitatively better to use the Manders' coefficients that separate the analysis as in case (c).

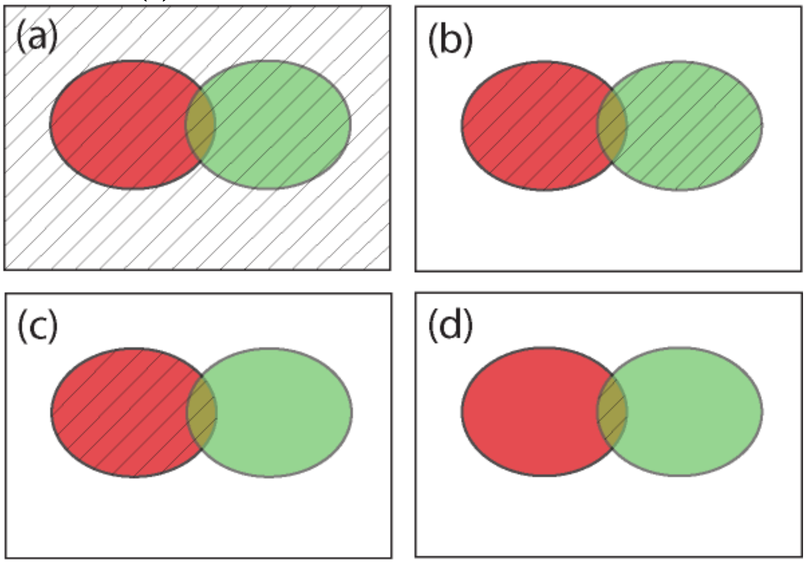

Figure 3 - Qualitative representation of an image with two channels, red and green, and different possible regions of interest. (a) The region of interest is the whole image. (b) The region of interest is defined by the pixels where the signal of the red or green channel is higher than zero. (c) The region of interest is defined by the pixels where one of the signals is greater than zero, in this case the red signal. (d) The region of interest is defined as the pixels where both channels have a signal greater than zero.

Although the Manders' coefficients have been specially designed to evaluate the degree of colocalization for case (c), the H_{coeff}, and the $\boldsymbol{R}$ can also be modified for that purpose and they can even be used for case (d).

To estimate the colocalization or correlation in case (c), the $\boldsymbol{R}$ can be divided in the following two coefficients,

$$\boldsymbol{R}_G = \frac{\sum_{i=1}^{N_{Gpix}} \left(Ir_i - \langle Ir \rangle_{Gpix}\right)\left(Ig_i - \langle Ig \rangle_{Gpix}\right)}{\sqrt{\left(\sum_{i=1}^{N_{Gpix}} \left(Ir_i - \langle Ir \rangle_{Gpix}\right)^2\right)\left(\sum_{i=1}^{N_{Gpix}} \left(Ig_i - \langle Ig \rangle_{Gpix}\right)^2\right)}},$$

$$\boldsymbol{R}_R = \frac{\sum_{i=1}^{N_R} \left(Ir_i - \langle Ir \rangle_{Rpix}\right)\left(Ig_i - \langle Ig \rangle_{Rpix}\right)}{\sqrt{\left(\sum_{i=1}^{N_R} \left(Ir_i - \langle Ir \rangle_{Rpix}\right)^2\right)\left(\sum_{i=1}^{N_R} \left(Ig_i - \langle Ig \rangle_{Rpix}\right)^2\right)}} \qquad (7)$$

Where the pixels considered for the evaluation of the $\boldsymbol{R_G}$ and the $\boldsymbol{R_R}$ are those pixels where the G and R signal are greater than zero respectively. And for case (d) the $\boldsymbol{R}$ would be,

$$\boldsymbol{R}_{RG} = \frac{\sum_{i=1}^{N_{RGpix}} \left(Ir_i - \langle Ir \rangle_{Gpix}\right)\left(Ig_i - \langle Ig \rangle_{Rpix}\right)}{\sqrt{\left(\sum_{i=1}^{N_{RGpix}} \left(Ir_i - \langle Ir \rangle_{Gpix}\right)^2\right)\left(\sum_{i=1}^{N_{RGpix}} \left(Ig_i - \langle Ig \rangle_{Rpix}\right)^2\right)}} \qquad (8)$$

Where the signal considered in the evaluation is only in the pixels where the signal in the R and G channels is greater than zero. Analogous relations can be derived for the Overlap coefficient by just removing the average over the intensities in the Pearson's equations.

We can obtain analogous equations for the H_{coeff} in case (c),

$$H_R = \frac{N_{pr}\sum_{i=1}^{N_p} Ir_i\ Ig_i}{\left(\sum_{i=1}^{N_p} Ir_i\right)\left(\sum_{i=1}^{N_p} Ig_{i,Coloc}\right)}, \quad H_G = \frac{N_{pg}\sum_{i=1}^{N_p} Ir_i\ Ig_i}{\left(\sum_{i=1}^{N_p} Ig_i\right)\left(\sum_{i=1}^{N_p} Ir_{i,Coloc}\right)}, \qquad (9)$$

and for case (d),

$$H_{RG} = \frac{N_{prg}\sum_{i=1}^{N_p} Ir_i\ Ig_i}{\left(\sum_{i=1}^{N_p} Ir_{i,coloc}\right)\left(\sum_{i=1}^{N_p} Ig_{i,coloc}\right)}. \qquad (10)$$

(ii) Colocalization equations for multicolor images

In some cases it is of interest to study simultaneously the binding or colocalization of more than two molecules labeled with different dyes. In this case it is possible to

generalize the previous coefficients to be used with more than two channels. The generalization of these coefficients to multicolor images can be done as follows,

$$H_{coeff} = \frac{N_p \sum_{i=1}^{N_p} \left(\prod_{\alpha} I\alpha_i \right)}{\prod_{\alpha} \left(\sum_{i=1}^{N_p} I\alpha_i \right)}. \qquad (13)$$

and

$$\boldsymbol{R} = \frac{\sum_{i=1}^{N_p} \prod_{\alpha} \left(I\alpha_i - \langle n\alpha \rangle \right)}{\prod_{\alpha} \sqrt{\left(\sum_{i=1}^{N_p} \left(I\alpha_i - \langle n\alpha \rangle \right)^2 \right)}} \qquad (14)$$

where the product, Π, expands over all the channels, α, under consideration.

	nr and ng	N_p	H_{coeff}	*R*	*Over*	M_R	M_G	H_R	H_G	P_R	P_G	H_{RG}	P_{RG}
**(a)**	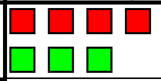	4	1	0	0.571	0.578	0.685	1	1	0	0	1	0
		9	1	0	0.317	0.297	0.375	1	1	0	0	1	0
**(b)**	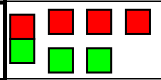	4	1.25	0.250	0.688	0.685	0.789	1.058	1.029	0.141	0.102	1.063	0.023
		9	1.666	0.278	0.508	0.473	0.583	1.049	1.023	0.109	0.072	1.039	0.003
**(c)**	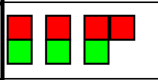	4	2	0.905	0.965	1	1	1.062	1.062	0.375	0.375	1.6	0.375
		9	3.666	0.934	0.953	1	1	1.032	1.032	0.197	0.197	1.64	0.197
**(d)**	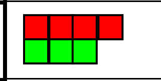	4	4	1	1	1	1	1	1	-	-	1	-
		9	9	1	1	1	1	1	1	-	-	1	-
**(e)**	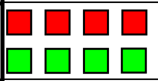	4	1	0	0.606	0.685	0.685	1	1	0	0	1	0
		9	1	0	0.351	0.375	0.375	1	1	0	0	1	0
**(f)**	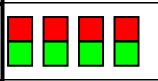	4	1.75	1	1	1	1	1.117	1.117	0.75	0.75	2.448	0.75
		9	3.	1	1	1	1	1.068	1.068	0.504	0.504	2.791	0.5048
**(g)**	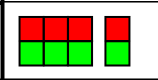	4	2.875	1	1	1	1	1.187	1.187	0.75	0.75	1.75	0.75
		9	6	1	1	1	1	1.222	1.222	0.888	0.888	1.888	0.8888
**(h)**	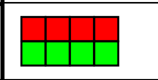	4	4	1	1	1	1	1	1	-	-	1	-
		9	9	1	1	1	1	1	1	-	-	1	-
**(i)**	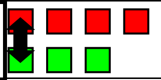	4	0.916	-0.084	0.532	0.542	0.648	0.979	0.989	-0.047	-0.034	0.956	-0.008
		9	0.916	-0.035	0.293	0.275	0.349	0.991	0.995	-0.013	-0.009	0.976	-0.000
**(j)**	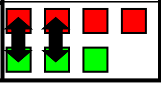	4	0.833	-0.166	0.493	0.507	0.613	0.953	0.976	-0.099	-0.072	0.901	-0.013
		9	0.833	-0.069	0.269	0.253	0.323	0.981	0.990	-0.028	-0.019	0.947	-0.000

Table 1 - Average colocalization coefficients for R (red) particles and G (green) particles located at 4 and 9 discrete positions. In (a)-(d), (i), (j) we consider 4 R and 3 G particles, and in (e)-(h) 4 R and 4 G particles. In (a) and (e) all the particles are randomly distributed. (b) One G particle is bounded to one R particle. (c) It is considered two R particles individually bound to two G

	A-A	*A-B*	*A-C*	*A-D*	*A-E*	*A-F*	*A-G*
R channel	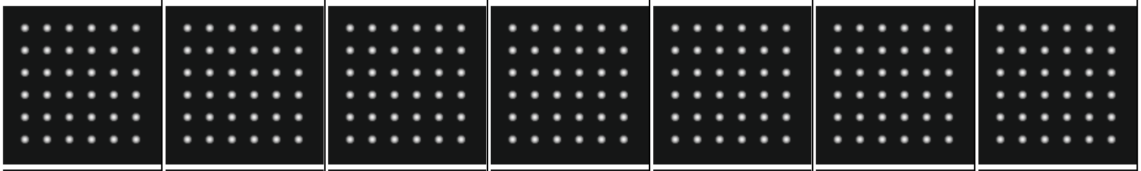						
G channel	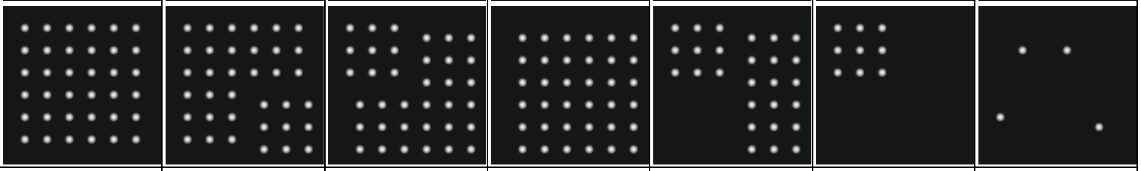						
H_{coeff}	13.4	10.023	3.34	0	4.455	13.4	10.023
P_{coeff}	1	0.7298	0.189	-0.081	0.2396	0.4855	0.235
Over	1	0.75	0.25	0	0.289	0.5	0.25
M_R	1	0.75	0.25	0	0.25	0.25	0.0833
M_G	1	0.75	0.25	0	0.333	1	0.75
H_R	1.065	1.065	1.065	-	1.065	1.065	1.065
H_G	1.065	1.065	1.065	-	1.065	1.065	1.065
P_R	1	0.392	0.141	-	0.1407	0.1407	0.074
P_G	1	0.3922	0.141	-	0.1715	1	0.3922
H_{RG}	1.065	1.065	1.065	-	1.065	1.065	1.065
P_{RG}	1	1	1	-	1	1	1

Table 2 - Colocalization coefficient results obtained after different combinations of abstract images composed of equal objects. In the first two rows are shown the pair of images taken as the red (R) and green (G) channel and in the rest of the rows the colocalization coefficients for each pair of images.

	A-Ah	*A-Bh*	*Bh-Bh*	*A-Ainv*	*Abin-AbinInv*	*A-ARd*	*A-Rd*
R channel	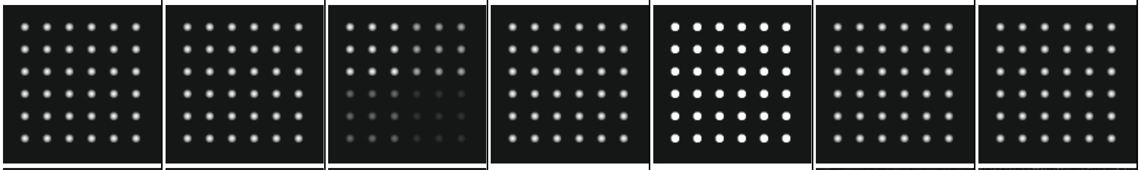						
G channel	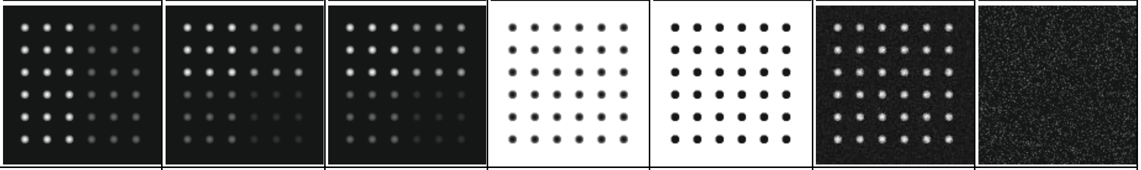						
H_{coeff}	13.4	13.4	16.038	0.311	0	12.895	1.0145
P_{coeff}	0.9449	0.9068	1	-1	-1	0.3459	0.0094
Over	0.9487	0.9129	1	0.0834	0	0.3588	0.0843
M_R	1	1	1	0.9896	0	0.9114	0.092
M_G	1	1	1	0.0283	0	0.9649	0.0883
H_R	1.0645	1.0645	1.2774	0.8731	-	1.0645	1.0054
H_G	1.0645	1.0645	1.2774	0.3138	-	7.5801	0.9845
P_R	0.5941	0.4822	1	-1	-	0.3199	0.0065

$\boldsymbol{P}_{G}$	0.5941	0.4822	1	-1	-	0.2696	-0.019
$\boldsymbol{H}_{RG}$	1.0645	1.0645	1.2774	0.8762	-	1.0427	0.9963
$\boldsymbol{P}_{RG}$	0.5941	0.482	1	-1	-	0.29	-0.056

Table 3 - Colocalization coefficient results obtained after different combinations of abstract images composed of different objects. In the first two rows are shown the pair of images taken as the red (R) and green (G) channel and in the rest of the rows the colocalization coefficients for each pair of images.
